# Influence of Dietary Algae Meal on Lipid Oxidation and Volatile Profile of Meat from Lambs with Competent Reticular Groove Reflex

**DOI:** 10.3390/foods11152193

**Published:** 2022-07-23

**Authors:** Carmen Avilés-Ramírez, Montserrat Vioque Amor, Oliva Polvillo Polo, Alberto Horcada, Pilar Gómez-Cortés, Miguel Ángel de la Fuente, Nieves Núñez-Sánchez, Andrés Luis Martínez Marín

**Affiliations:** 1 Departamento de Bromatología y Tecnología de los Alimentos, Universidad de Córdoba, Ctra. Madrid-Cádiz km 396, 14071 Córdoba, Spain; bt1viamm@uco.es; 2 Centro de Investigación, Tecnología e Innovación, Universidad de Sevilla, Avda. Reina Mercedes 4-B, 41012 Sevilla, Spain; oppolo@us.es; 3 Departamento de Agronomía, Escuela Técnica Superior de Ingeniería Agronómica, Universidad de Sevilla, 41013 Sevilla, Spain; albertohi@us.es; 4 Instituto de Investigación en Ciencias de la Alimentación (CIAL, CSIC-UAM), Nicolás Cabrera, 9, 28049 Madrid, Spain; p.g.cortes@csic.es (P.G.-C.); mafl@if.csic.es (M.Á.d.l.F.); 5 Departamento de Producción Animal, Universidad de Córdoba, Ctra. Madrid-Cádiz km 396, 14071 Córdoba, Spain; pa2nusan@uco.es (N.N.-S.); pa1martm@uco.es (A.L.M.M.)

**Keywords:** lamb, meat, marine algae, aroma, flavour, volatile organic compounds

## Abstract

Dietary lipid sources influence intramuscular fatty acid composition, which in turn may affect the volatile profile of meat. The aim of this work was to investigate the effects of marine algae supplementation (*Aurantiochytrium limacinum*) on volatile compounds of cooked lamb meat. Forty-eight lambs with 42 days of age were divided into three groups: lambs fed a conventional diet without algae meal supplementation (NOALG), lambs with competent reticular groove reflex (RGR) fed the same diet supplemented with 2.5% marine algae meal mixed in the concentrate (ALGCON), and lambs with competent RGR, receiving the same diet and fed with 2.5% marine algae meal in a milk replacer to bypass the rumen (ALGMILK). Lipid and protein oxidation in raw meat was assessed and volatile compounds in grilled meat were determined. The highest and lowest lipid oxidations were observed in the ALGMILK and NOALG groups, respectively. Protein oxidation was unaffected. Out of 56 identified compounds, 12 volatiles significantly increased in both algae groups and 6 of them exclusively in the ALGCON treatment. Algae meal supplementation and its form of administration, either protected or not from rumen degradation, are important factors to consider in lipid oxidation and the aromatic profile of lamb meat.

## 1. Introduction

Lamb meat is highly appreciated in certain Mediterranean countries and its characteristic flavour is closely related to its acceptance by consumers [1]. Aroma is the main determinant of meat flavour and derives from volatiles produced as a result of thermally induced reactions of non-volatile components during cooking. Such reactions include lipid oxidation, Maillard and Strecker reactions, the interaction of lipid oxidation products with Maillard’s reaction products, and vitamin degradation [2,3].

Together with low molecular weight water-soluble compounds, fat constitutes the most important precursor of cooked meat flavour [3]. Polyunsaturated fatty acids (PUFA), which are preferentially deposited in the muscle cell phospholipids, have a key role in the aroma of meat due to their tendency to oxidise during cooking [4]. Most of the carbonyl volatile compounds are generated from the oxidation of unsaturated fatty acids (FA) and can be intense aroma compounds [5]. Since the feeding system is a major determinant of the FA profile of intramuscular fat [6], it has an important role in lamb meat aroma [7,8].

Marine microalgae, such as *Aurantiochytrium* sp. and *Isochrysis* sp., are strong candidates as dietary supplements due to their improvement in the nutritional value of lamb meat in terms of health-claimable omega-3 FA [9]. In this line, a successful alternative to enhance the omega-3 FA content in ruminant meat is to foster the reticular groove reflex (RGR) of the newborn animal into adulthood and use it to feed emulsified lipid sources directly into the abomasum, thus bypassing the rumen. This approach to protect dietary PUFA against rumen biohydrogenation was recently researched in lambs by Gómez-Cortés et al. [10]. Feeding marine microalgae to RGR competent feedlot lambs allowed to obtain meat with greater contents of eicosapentaenoic acid (EPA) and docosahexaenoic acid (DHA). Furthermore, this nutritional value enhancement took place without adverse effects on growth performance, carcass traits, and meat characteristics [11].

Nevertheless, the effects of diets containing microalgae on lamb meat volatile profiles have been very scarcely examined [12]. Therefore, the aim of the present work was to investigate the effects of marine microalgae, either fed in the concentrate or in a milk replacer to bypass the rumen, on lipid and protein oxidation and the volatile profile of meat from lambs with competent RGR.

## 2. Materials and Methods

### 2.1. Experimental Design

All animal procedures used in this experiment were carried out following the Spanish regulations on protection of experimental animals (authorization no. 04/05/2018/074). Details on the experimental design and diet composition have been published in a previous work [10]. In brief, 48 lambs from Manchega breed (42 days old and 11.6 ± 1.67 kg of body weight, BW) were allocated in pairs in 24 pens (Animal Production Department, University of Córdoba, Córdoba, Spain). Pens were distributed in 8 blocks according to average BW and allocated randomly to one of three dietary treatment groups (16 animals per treatment): (i) the control group, which was fed a typical pelleted concentrate and did not consume algae meal or milk replacer (NOALG), (ii) the algae meal in concentrate group, which received the same concentrate as NOALG but mixed with 2.5% algae meal (*Aurantiochytrium limacinum*; Forplus, Alltech Spain, Guadalajara, Spain) plus 250 mL daily of milk replacer in a single feeding (ALGCON), and (iii) the algae meal in milk replacer group, which received the same concentrate as NOALG plus 250 mL daily of milk replacer supplemented with algae meal in a single feeding (ALGMILK).

Feeding milk replacer daily ensured that lambs in both ALGCON and ALGMILK experimental groups maintained the competency of the RGR. When the average BW of the lambs reached ~25 kg (49 days on fattening), animals were transported in an adequately conditioned vehicle to an officially approved slaughterhouse. Lambs were stunned, slaughtered, and dressed using standard procedures. All carcasses (10.8 ± 1.1 kg) were chilled at 4.0 °C for 24 h in a commercial chiller. Then, the carcasses were transferred to the Laboratory of Meat Quality at the Animal Production building of the University of Córdoba (Córdoba, Spain) respecting the cold chain. In the laboratory, the *Longissimus thoracis* muscle from the right carcass was vacuum-packed and samples for oxidation and volatile analyses were obtained after 6 days of ageing at 4 °C.

### 2.2. Lipid and Protein Oxidation Measurement

To quantify the extension of lipid oxidation, thiobarbituric acid reacting substances (TBARS) were measured by Tarladgis et al. method [13], as described by Avilés et al. [14]. Briefly, 10 g of each meat sample was blended in 50 mL of distilled water for 120 s at 12,000 rpm (Ultra-Turrax T25, IKA Werbe GmbH & Co., KG, Staufen, Germany). The mixture was transferred to a Kjeldahl flask containing a small amount of silicone antifoam by washing with distilled water, 4N HCl, and an antioxidant solution. The flasks were then heated until 50 mL of distillate were collected. Following, 5 mL of the distillate were mixed with 5 mL of TBARS in falcon-type tubes, shaken to mix the contents, and immersed in a boiling water bath for 35 min. Finally, the tubes were cooled under tap water for 10 min, and the absorbance was measured in a spectrophotometer (Helios spectrophotometer, Thermo Scientific, Bremen, Germany) at 530 nm. The 1,1,3,3-tetra-ethoxypropane standard curve was used for calculating the TBARS concentration and the results were expressed as mg of malonaldehyde (MDA) per kg of meat.

Protein oxidation was measured by an estimation of carbonyl groups formed, according to the methodology described by Mercier et al. [15] with minor modifications. Ten ml of KCl 0.15M was added to 1 g of sample and homogenized (Ultra-Turrax T25, IKA Werbe GmbH & Co., KG, Staufen, Germany). Two aliquots of 0.5 mL of the homogenate were treated with 20% trichloroacetic acid (TCA) and centrifuged at 2500 rpm for 10 min. One pellet was treated with 1ml of 2N HCl and the other with an equal volume of 0.2% (*w*/*v*) 2,4-dinitrophenylhydrazine (DNPH) in 2N HCl. Both samples were incubated for 1 h at room temperature before samples were treated again with 20% TCA as previously. Then, the pellets were washed with 1 mL of ethanol:ethyl acetate (1:1), shaken, and centrifuged for 5 min at 2500 rpm; this procedure was repeated twice. Finally, the pellets were re-suspended in 2 mL of 6 M guanidine HCl with 20 mM Na_3_PO_4_ buffer pH 6.5, shaken, and centrifuged for 2 min at 2500 rpm. BSA in 6 M guanidine was used as a standard to measure protein concentration at 280 nm. Carbonyl concentration expressed as nmol of DNPH fixed per mg of protein was measured on the treated sample by measuring DNPH incorporated on the basis of an absorption of 21.0 mM^−1^ cm^−1^ at 370 nm for protein hydrazones.

### 2.3. Volatile Organic Compound Analysis

A steak of ~1.8 cm of thickness was separated from each sample and cooked on a double-clamp electric grill (Jatta Electro, GR266 1000W, Abadiano, Vizcaya, Spain) at 170 °C until a core temperature of 70 °C. Each sample was positioned in the middle of the grilling tray to ensure uniform grilling. Meat and fat were manually trimmed just after cooking and 10 g of sample were placed in a headspace vial (Tekmar, 100 mL). Volatile compounds were extracted by headspace solid-phase microextraction, separated by gas chromatography and their mass spectra generated in the same equipment and conditions as recently described by Gutiérrez-Peña et al. [16]. The peak area of the volatile compounds was integrated to estimate their relative abundance. A series of n-alkanes (C5–C18, HP5080-8768) was analysed under the same conditions to obtain the linear retention index (LRI) value for each volatile compound. The tentative identification of each compound was done by comparing their mass spectra with those from the mass spectra library of the National Institute of Standards and Technology (NIST vers. 2.0, Gaithersburg, MD, USA) or those previously published in the literature.

### 2.4. Statistical Analysis

All statistical analyses were carried out with XLSTAT 2020 (Addinsoft Inc., New York, NY, USA). Each sample was an experimental unit. Data of volatile compounds were expressed as area units (AU) calculated as the log10 transformation of the peak area mean values (from duplicated samples). Lipid oxidation, protein oxidation, and volatile compound data were analysed by ANOVA as a completely randomized design, according to the following model: Yij = μ + Ti + eij, where Yij is the dependent variable, μ is the overall mean, Ti is the fixed effect treatment (NOALG, ALGCON, ALGMILK), and eij is the residual error. When the model was significant, differences between means were assessed by Tukey’s test option in the statistical software. Data were also analysed by principal component analysis (PCA) to obtain an overview of dietary treatments and their indicative volatile profile. The principal components (PC) were orthogonally rotated with the Varimax option and Kaiser normalization to improve readability. According to the interpretability criteria, only PC1 and PC2 were retained [17]. The PC scores were further investigated by ANOVA, using treatment as fixed effect. Statistical significance was declared at *p* < 0.05.

## 3. Results and Discussion

Lipid oxidation differed (*p* < 0.05) between all treatments. It was higher in the ALGMILK treatment followed by the ALGCON treatment and lower in the NOALG treatment (Table 1). The richness of long chain omega-3 PUFA in the intramuscular fat of the marine algae-supplemented meat samples as compared to the control samples (4.45 ± 1.48% vs. 1.45 ± 0.45% total FA, respectively) would explain their proneness to oxidation [10,18,19]. Oxidation changes the colour and lipid composition, which leads to the deterioration of visual appearance, undesirable flavours, and rancidity in meat. Those modifications may shorten meat shelf life [20]. Nevertheless, the lipid oxidation values were below the proposed threshold of 2.3 mg MDA per kg of meat for the meat samples to be recognized as rancid [21]. In contrast to Karabagias [22], hexanal to nonanal, heptanal to nonanal, octanal to nonanal ratios, along with the sum of hexanal, heptanal, and octanal to nonanal ratio, were not correlated (*p* > 0.05) with the degree of lipid oxidation (data not shown). Again, no differences (*p* > 0.05) were found in protein oxidation (Table 1). Results from previously published articles suggest that diet has no effect on the protein oxidation of lamb meat despite differences in total omega-3 FA contents [23,24].

A total of 56 volatile organic compounds belonging to eleven chemical families (18 aldehydes, 12 alcohols, 8 ketones, 3 organic acids, 2 aromatic hydrocarbons, 3 aliphatic hydrocarbons, 2 esters, 3 lactones, 2 terpenoids, 2 furans, and 1 pyrazine) were detected in meat (Table 2). That figure is more than double that found in light lambs fed with a commercial concentrate with a similar composition to that of the present work [25], but much lower than that found by Elmore et al. [12] in meat from lambs supplemented with several fat sources. Analytical differences might account for such discrepancies. Algae meal supplementation in the concentrate promoted the highest concentration of volatile compounds and the most variable profile. The ALGCON treatment was richer (*p* < 0.05) in total aldehydes, alcohols, ketones, aliphatic hydrocarbons, lactones, and pyrazines. These results suggest an interaction of marine algae supplementation with its way of administration. In contrast to marine algae supplied in the milk replacer, marine algae supplied in the concentrate may interact with rumen microorganisms and the resulting increased absorption and deposition of FA and other compounds from microbial origin in tissue lipids could favour the diversity of volatile compounds in meat [7].

Lipid oxidation, either during meat storage or as a result of cooking, together with the Maillard reaction during cooking, are the main factors related to the typical aroma of meat [2,3]. Aldehydes, alcohols, and ketones were the most abundant volatiles detected in the present work, and along with other minor groups such as hydrocarbons and lactones, are known to be related to lipid oxidation [2,3]. Volatile compounds related to the Maillard reaction [2,3], such as esters, pyrazines, or furans, were a minority (Table 2). The overall scarcity of Maillard reaction-derived compounds in the meat samples might be related to the fact that thin steaks and low grilling temperatures were used. It would favour the formation of volatiles from lipid oxidation at the expense of Maillard reaction volatiles [26].

Aldehydes, alcohols, and ketones were the most abundant volatiles in all samples, accounting for more than 70% of the total volatiles detected. It has been observed that these compounds show a higher relative abundance in lamb meat regardless of if the animals are reared on pastures or fed concentrates indoor [27]. Aldehydes and ketones were found in greater amounts (*p* < 0.05) in the ALGCON treatment (Table 2). Moreover, the sum of the aldehydes and ketones was greater in the ALGCON treatment, when compared to ALGMILK, which in turn has a higher value than the NOALG treatment (*p* < 0.05). Since those volatile compounds are negatively correlated with the lamb flavour of light lambs reared under intensive conditions [28], it might be expected that meat from the algae-supplemented lambs would have a less intense lamb flavour. Volatile compounds associated with mutton odour, such as 4-methyloctanoic and 4-methylnonanoic acids, and to animal odour or barnyard odour, such as 3-methylindole, ethyl acetate, 2-methylpropanal, and methional [29,30,31] were not detected in the present work, which might be related to the young age of the animals and the rearing conditions [32,33,34].

Up to 20 volatile compounds were affected (*p* < 0.05) by diet: two aldehydes, five alcohols, one ketone, one aromatic hydrocarbon, two aliphatic hydrocarbons, two esters, three lactones, two terpenoids, one furan, and one pyrazine (Table 2). Six out of the 20 aforementioned compounds, including pentanal, 1-penten-3-ol, (E)-2-octen-1-ol, 2-ethylhexan-1-ol, γ-butyrolactone, and 4-methyl-2-propyl furan, were only detected in the marine algae-supplemented treatments. In partial agreement with these results, the first three compounds above have been previously related in lamb meat to the consumption during the fattening period of a long chain omega-3-enriched diet by the supplementation of a combination of fish oil and algae meal [12]. On the other hand, marine oil degradation is characterized by high levels of 1-penten-3-ol, a compound that derives exclusively from the oxidation of the omega-3 terminal end of EPA and DHA [35]. To the best of our knowledge, the relationship of 2-ethyl-hexan-1-ol, γ-butyrolactone, and 4-methyl-2-propyl furan in lamb meat with the consumption of diets supplemented with algae meal or enriched in long chain omega-3 FA has not been yet reported. The 2-ethyl-hexan-1-ol is increased in meat from beef cattle supplemented with 200 mg/d of *Schizochytrium* sp. powder [36]. Furthermore, it has been observed that meat from pasture-fed ruminants has a greater amount of 2-ethyl-hexan-1-ol and γ-butyrolactone than meat from grain-fed ruminants in concurrence with higher contents of omega-3 PUFA in intramuscular fat [26,37].

Six out of the 20 compounds that showed significant differences between diets, including acetophenone, pentadecane, methyl 9,12-hexadecadienoate, octalactone, phytol, and trimethylpyrazine, were only found in the ALGCON treatment (Table 2). Acetophenone has been reported in perirenal and heart fat of pasture-fed lambs [38], but its presence in lamb meat has not been found to be related to long chain omega-3-enriched diets in previous studies [39], while pentadecane is clearly associated with the consumption of such omega-3 diets [12,39]. The fact that pentadecane did not increase in ALGMILK samples might suggest that an interaction between marine algae oil and rumen microorganisms is needed to promote its presence in meat.

No information regarding the relationship between methyl 9,12-hexadecadienoate, octalactone, phytol, and trimethylpirazyne with feeding lambs diets supplemented with algae meal or enriched in long chain omega-3 PUFA has been previously reported. Octalactone in lamb meat has been found not to be affected by replacing a 100% silage diet with a 100% concentrate diet nor by completely substituting dehydrated alfalfa for cardoon meal [40,41]. Phytol in beef meat has been negatively related to grain feeding in drylot after grazing on pastures [42,43]. Moreover, phytol was found in a greater amount in loin fat of a clover-finished lamb than in that of a maize-finished lamb [44]. Trimethylpyrazine has been well described in lamb meat [45,46], but it seems not to be related to diet characteristics [16,47,48].

Four out of the 20 compounds that showed significant differences between diets, including ethanol, methyl (4E)-4-octadecenoate, γ-dodecalactone, and farnesol, were only found in the NOALG treatment (Table 2). Contrary to our results, ethanol has been reported in a higher amount in meat from pasture-fed lambs as well as in beef cattle fed 100 mg/d of *Schizochytrium* sp. powder, i.e., from animals with intramuscular fat rich in long chain omega-3 FA [27,36]. The γ-dodecalactone has been reported in greater amounts in loin fat of a clover-finished lamb than in that of a maize-finished lamb [44]. Regarding other volatiles in lamb meat such as 4-methyl-2-propyl furan, methyl 9,12-hexadecadienoate, methyl (4E)-4-octadecenoate and farnesol, the information available in the literature is very scarce.

**Figure 1:**
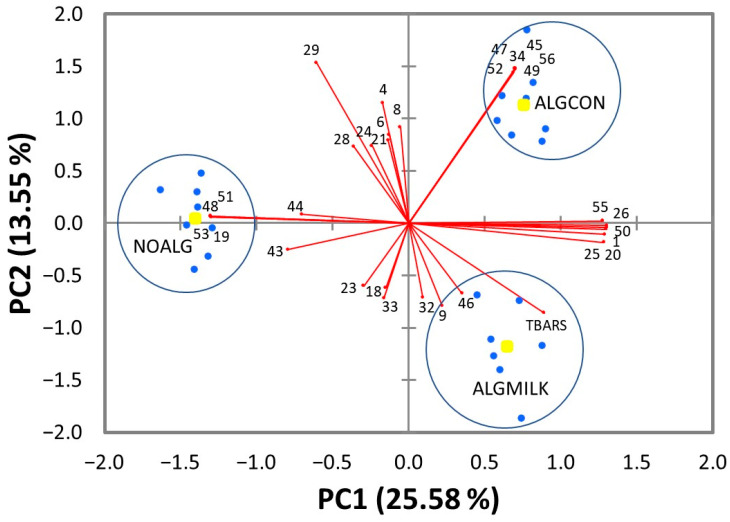
Principal component analysis of volatile compounds and thiobarbituric acid reacting substances (TBARS) in meat from lambs fed a control diet (NOALG) and lambs with competent reticular groove reflex fed the control diet supplemented with 2.5% marine algae meal mixed in the concentrate (ALGCON) or in a milk replacer (ALGMILK). Only selected volatile compounds with loadings greater than ±0.6 are shown. Numbers refer to the ordinal number assigned to each volatile compound in Table 2. Blue circles represent the position of the samples in the principal component space and yellow circles are the centroids of each group.

A PCA was carried out in order to differentiate experimental treatments according to their volatile profile and lipid oxidation parameters. Two significant PC were retained, which described 25.58% (PC1) and 13.55% (PC2) of the total variance. The score plot (Figure 1) showed three well-defined clusters of observations differentiating the three experimental treatments. In general terms, meat samples were discriminated according to marine algae supplementation by PC1 (negative for NOALG and positive for ALGCON and ALGMILK treatments) and according to its presence in the diet and via its administration by PC2 (near to zero, positive and negative for the NOALG, ALGCON, ALGMILK treatments, respectively). Pentanal, 1-penten-3-ol, 2-ethylhexan-1-ol, (E)-2-octen-1-ol, octalactone, and 4-methyl-2-propyl furan had high and positive loadings in PC1, whereas ethanol, methyl (4E)-4-octadecenoate, γ-dodecalactone, and farnesol had high and negative loadings. Thus, the former group was associated with marine algae supplementation whereas the latter group was characteristic of an absence of such supplementation.

Acetophenone, pentadecane, methyl 9,12-hexadecadienoate, γ-butyrolactone, phytol, and trimethylpyrazine had positive loadings in PC1 and PC2 and thus they were associated with the ALGCON treatment. Interestingly, γ-butyrolactone, which did not differ between the marine algae-supplemented treatments (Table 2), was positively related with algae supplementation via concentrate and negatively related with its supplementation via RGR in the milk replacer. The (E)-2-nonenal, 3-methyl-2-octanone, hexadecane, and TBARS had positive loadings in PC1 and negative loadings in PC2, hence they were closely related with the ALGMILK treatment. The ANOVA of the PC scores (Table 3) confirmed that PC1 effectively separated the unsupplemented treatment from the supplemented ones (*p* < 0.05) and that PC2 discriminated all three treatments (*p* < 0.05).

## 4. Conclusions

Marine algae meal supplementation increased the lipid oxidation of lamb meat during ageing and modified its volatile profile when grilled. Lipid oxidation was enhanced by feeding the algae meal in the milk replacer, thus bypassing rumen degradation, while feeding the algae meal in the concentrate provided a richer and more varied profile of volatile compounds. Several compounds were found to be characteristic of algae meal supplementation and the presence of some of them was affected by the way the microalgae was supplied. Overall, algae meal supplements and their form of administration, either protected or not from rumen interactions, substantially contribute to the aromatic profile of lamb meat.

## Figures and Tables

**Table 1:** Lipid and protein oxidation in meat from lambs fed a control diet (NOALG) and lambs with competent reticular groove reflex fed the control diet supplemented with 2.5% of marine algae meal mixed in the concentrate (ALGCON) or in a milk replacer (ALGMILK).

Oxidation ^1^	Treatment			SEM	*p*-Value ^2^
	NOALG	ALGCON	ALGMILK		
Lipid, mg MDA/kg meat	0.221 ^c^	0.509 ^b^	0.794 ^a^	0.0625	***
Protein, nmol DNPH/mg protein	2.441	2.601	2.778	0.0961	ns

^1^ MDA: malonaldehyde. DNHP: 2,4-dinitrophenylhydrazine. ^2^ *** = *p* < 0.001, ns = *p* ≥ 0.05. ^a,b,c^ Means without a common superscript letter are statistically different by Tukey test at *p* < 0.05.

**Table 2:** Volatile profile of grilled meat from lambs fed a control diet (NOALG) and lambs with competent reticular groove reflex fed the control diet supplemented with 2.5% of marine algae meal mixed in the concentrate (ALGCON) or in a milk replacer (ALGMILK).

Volatile Compounds (Area Units)		LRI ^1^	Method of Identification		Treatment			SEM	*p*-Value ^4^
			RI ^2^	MS ^3^	NOALG	ALGCON	ALGMILK		
	**Aldehydes**								
1	Pentanal	989	×	×	0.00 ^b^	10.22 ^a^	10.11 ^a^	1.000	***
2	Hexanal	1092	×	×	10.25	10.86	10.51	0.175	ns
3	Heptanal	1193	×	×	9.22	9.58	9.66	0.210	ns
4	Octanal	1300	×	×	9.10 ^ab^	9.52 ^a^	8.31 ^b^	0.177	*
5	Nonanal	1409	×	×	9.38	9.56	9.26	0.218	ns
6	(E)-Oct-2-enal	1446	×	×	9.23	9.40	8.69	0.145	ns
7	(E,E)-2,4-Heptadienal	1514	×	×	7.91	8.92	8.55	0.235	ns
8	Benzaldehyde	1549	×	×	9.33	9.65	8.72	0.203	ns
9	(E)-2-Nonenal	1554	×	×	8.45	8.48	9.02	0.180	ns
10	(Z)-2-Decenal	1664			9.21	8.38	8.93	0.235	ns
11	Benzeneacetaldehyde	1670		×	8.39	8.97	8.70	0.153	ns
12	2,4-Nonadienal	1724	×	×	8.91	8.58	8.28	0.132	ns
13	2-Undecenal	1773	×	×	8.52	8.32	8.56	0.199	ns
14	(E,E)-2,4-Decadienal	1788	×	×	9.35	8.88	8.92	0.162	ns
15	2,4-Undecadienal	1830			9.40	9.18	8.75	0.183	ns
16	Tetradecanal	1921	×	×	9.12	9.11	8.88	0.127	ns
17	Pentadecanal	2007	×	×	9.08	9.21	8.96	0.162	ns
18	Hexadecanal	2081	×	×	9.03	8.66	9.01	0.164	ns
	Total aldehydes				144.85 ^c^	156.83 ^a^	152.74 ^b^	1.180	***
	**Alcohols**								
19	Ethanol	941	×	×	10.63 ^a^	0.00 ^b^	0.00 ^b^	1.045	***
20	1-Penten-3-ol	1176	×	×	0.00 ^b^	8.89 ^a^	9.32 ^a^	0.904	***
21	1-Pentanol	1263	×	×	9.49	9.66	9.07	0.182	ns
22	1-Hexanol	1365	×	×	9.56	9.18	8.56	0.229	ns
23	1-Octen-3-ol	1462	×	×	10.14	9.26	9.96	0.230	ns
24	1-Heptanol	1468	×	×	9.42	9.36	8.72	0.208	ns
25	2-Ethylhexan-1-ol	1499	×	×	0.00 ^b^	8.75 ^a^	9.57 ^a^	0.913	***
26	(E)-2-Octen-1-ol	1564	×	×	0.00 ^b^	9.36 ^a^	9.12 ^a^	0.909	***
27	1-Octanol	1571	×	×	9.46	9.06	9.37	0.155	ns
28	(Z)-2-Nonen-1-ol	1628		×	9.47	9.23	8.53	0.185	ns
29	2-Dodecen-1-ol	1766	×	×	9.46 ^a^	9.42 ^a^	0.00 ^b^	0.928	***
30	Benzyl alcohol	1890	×	×	8.64	8.42	8.31	0.254	ns
	Total alcohols				86.27 ^b^	100.57 ^a^	90.53 ^b^	1.426	***
	**Ketones**								
31	3-Hydroxy-2-butanone	1304	×	×	9.33	9.10	9.50	0.193	ns
32	3-Methyl-2-octanone	1336		×	9.06	8.81	9.53	0.144	ns
33	2,3-Octanedione	1338	×	×	9.36	8.79	9.51	0.173	ns
34	Acetophenone	1677	×	×	0.00 ^b^	8.09 ^a^	0.00 ^b^	0.810	***
35	9-Decen-2-one	1823		×	8.08	8.64	8.69	0.184	ns
36	2-Tetradecanone	2000	×	×	9.17	8.49	8.68	0.210	ns
37	2-Pentadecanone	2074	×	×	8.78	8.70	8.90	0.177	ns
38	2-Hexadecanone	2146	×	×	8.64	8.75	9.00	0.199	ns
	Total ketones				62.41 ^b^	69.37 ^a^	63.79 ^b^	0.881	***
	**Organic acids**								
39	Acetic acid	1483	×	×	9.38	9.39	8.49	0.192	ns
40	Hexanoic acid	1863	×	×	8.22	8.39	8.06	0.235	ns
41	Octanoic acid	2033	×	×	8.54	8.01	8.16	0.181	ns
	Total organic acids				26.14	25.79	24.71	0.392	ns
	**Aromatic hydrocarbons**								
42	Toluene	1056	×	×	10.42	9.23	9.66	0.237	ns
43	Styrene	1270		×	8.86 ^a^	7.96 ^b^	8.15 ^b^	0.136	*
	Total aromatic hydrocarbons				19.28 ^a^	17.18 ^b^	17.81 ^b^	0.279	**
	**Aliphatic hydrocarbons**								
44	Tetradecane	1407	×	×	9.84 ^a^	8.90 ^ab^	8.67 ^b^	0.190	*
45	Pentadecane	1505	×	×	0.00 ^b^	9.26 ^a^	0.00^b^	0.910	***
46	Hexadecane	1606	×	×	8.42	8.63	8.99	0.140	ns
	Total aliphatic hydrocarbons				18.27 ^b^	26.79 ^a^	17.66 ^b^	0.896	***
	**Esters**								
47	Methyl 9,12-hexadecadienoate	1820		×	0.00 ^b^	8.26 ^a^	0.00 ^b^	0.818	***
48	Methyl (4E)-4-octadecenoate	2136		×	10.21 ^a^	0.00 ^b^	0.00 ^b^	1.004	***
	Total esters				10.21 ^a^	8.26 ^b^	0.00 ^c^	0.929	**
	**Lactones**								
49	γ-Butyrolactone	1662	×	×	0.00 ^b^	9.63 ^a^	9.67 ^a^	0.943	***
50	Octalactone	2093	×	×	0.00 ^b^	9.59 ^a^	0.00 ^b^	0.950	***
51	γ-Dodecalactone	2249	×	×	9.94 ^a^	0.00 ^b^	0.00 ^b^	0.977	***
	Total lactones				9.94 ^b^	19.22 ^a^	9.67 ^b^	0.928	***
	**Terpenoids**								
52	Phytol	2022		×	0.00 ^b^	9.29 ^a^	0.00 ^b^	0.913	***
53	Farnesol	2222		×	10.22 ^a^	0.00 ^b^	0.00 ^b^	1.008	***
	Total terpenoids				10.22 ^a^	9.29 ^b^	0.00 ^c^	0.965	***
	**Furans**								
54	2-Pentylfuran	1237	×	×	9.20	8.67	9.00	0.186	ns
55	4-Methyl-2-propyl furan	1590		×	0.00 ^b^	8.72 ^a^	8.45 ^a^	0.860	***
	Total furans				9.20 ^b^	17.38 ^a^	17.45 ^a^	0.839	***
	**Pyrazines**								
56	Trimethylpyrazine	1419	×	×	0.00 ^b^	8.75 ^a^	0.00 ^b^	0.861	***

^1^ LRI: Linear retention index. ^2^ RI: identification based on published retention index (RI) values. ^3^ MS: identification based on published mass spectra (MS). ^4^ * = *p* < 0.05, ** = *p* < 0.01, *** = *p* < 0.001, ns = *p* ≥ 0.05. ^a,b,c^. Means without a common superscript letter are statistically different by Tukey test at *p* < 0.05.

**Table 3:** Results of ANOVA of the principal component scores obtained from the principal components analysis shown in Figure 1.

Principal Component	Treatment ^1^			SEM	*p*-Value ^2^
	NOALG	ALGCON	ALGMILK		
PC1	−1.40 ^b^	0.76 ^a^	0.65 ^a^	0.209	***
PC2	0.05 ^b^	1.13 ^a^	−1.18 ^c^	0.209	***

^1^ NOALG: control diet (conventional lambs). ALGCON: control diet supplemented with 2.5% of marine algae meal mixed in the concentrate (lambs with competent reticular groove reflex). ALGMILK: control diet supplemented with 2.5% of marine algae meal in a milk replacer (lambs with competent reticular groove reflex). ^2^ *** = *p* < 0.001. ^a,b,c^. Means without a common superscript letter are statistically different by Tukey test at *p* < 0.05.

## Data Availability

The data presented in this study are available in the article.

## References

[1] Garmyn A. (2020). Consumer Preferences and Acceptance of Meat Products. Foods.

[2] Khan M.I., Jo C., Tariq M.R. (2015). Meat flavor precursors and factors influencing flavor precursors—A systematic review. Meat Sci..

[3] Resconi V.C., Escudero A., Campo M.M. (2013). The Development of Aromas in Ruminant Meat. Molecules.

[4] Nute G.R., Richardson R.I., Wood J.D., Hughes S.I., Wilkinson R.G., Cooper S.L., Sinclair L.A. (2007). Effect of dietary oil source on the flavour and the colour and lipid stability of lamb meat. Meat Sci..

[5] Bravo-Lamas L., Barrón L.J.R., Farmer L., Aldai N. (2018). Fatty acid composition of intramuscular fat and odour-active compounds of lamb commercialized in northern Spain. Meat Sci..

[6] Chikwanha O.C., Vahmani P., Muchenje V., Dugan M.E.R., Mapiye C. (2018). Nutritional enhancement of sheep meat fatty acid profile for human health and wellbeing. Food Res. Int..

[7] Vasta V., Priolo A. (2006). Ruminant fat volatiles as affected by diet. A review. Meat Sci..

[8] Watkins P.J., Frank D., Singh T.K., Young O.A., Warner R.D. (2013). Sheepmeat Flavor and the Effect of Different Feeding Systems: A Review. J. Agric. Food Chem..

[9] Alvarenga T.I.R.C., Chen Y., Furusho-Garcia I.F., Perez J.R.O., Hopkins D.L. (2015). Manipulation of Omega-3 PUFAs in Lamb: Phenotypic and Genotypic Views. Compr. Rev. Food Sci. Food Saf..

[10] Gómez-Cortés P., de la Fuente M.A., Peña Blanco F., Núñez-Sánchez N., Requena Domenech F., Martínez Marín A.L. (2021). Feeding Algae Meal to Feedlot Lambs with Competent Reticular Groove Reflex Increases Omega-3 Fatty Acids in Meat. Foods.

[11] Núñez-Sánchez N., Avilés Ramírez C., Peña Blanco F., Gómez-Cortés P., de la Fuente M.A., Vioque Amor M., Horcada Ibáñez A., Martínez Marín A.L. (2021). Effects of Algae Meal Supplementation in Feedlot Lambs with Competent Reticular Groove Reflex on Growth Performance, Carcass Traits and Meat Characteristics. Foods.

[12] Elmore J.S., Cooper S.L., Enser M., Mottram D.S.M.A., Sinclair L.A., Wilkinson R.G., Wood J.D. (2005). Dietary manipulation of fatty acid composition in lamb meat and its effect on the volatile aroma compounds of grilled lamb. Meat Sci..

[13] Tarladgis B.G., Watts B.M., Younathan M.T., Dugan L. (1960). A distillation method for the quantitative determination of malonaldehyde in rancid foods. J. Am. Oil Chem. Soc..

[14] Avilés Ramírez C., Peña Blanco F., Horcada Ibáñez A., Nuñez Sánchez N., Requena Domenech F., Guzmán Medina P., Martínez Marín A.L. (2019). Effects of concentrates rich in by-products on growth performance, carcass characteristics and meat quality traits of light lambs. Anim. Prod. Sci..

[15] Mercier Y., Gatellier P., Renerre M. (2004). Lipid and protein oxidation in vitro, and antioxidant potential in meat from Charolais cows finished on pasture or mixed diet. Meat Sci..

[16] Gutiérrez-Peña R., García-Infanter M., Delgado-Pertíñez M., Guzmán J.L., Zarazaga L.A., Simal S., Horcada A. (2022). Organoleptic and Nutritional Traits of Lambs from Spanish Mediterranean Islands Raised under a Traditional Production System. Foods.

[17] Suhr D.D. Principal Component Analysis vs. Exploratory Factor Analysis. https://support.sas.com/resources/papers/proceedings/proceedings/sugi30/203-30.pdf.

[18] de La Fuente-Vazquez J., Díaz-Díaz-Chirón M.T., Pérez-Marcos C., Cañeque-Martínez V., Sánchez-González C.I., Alvarez-Acero I., Fernández-Bermejo C., Rivas-Cañedo A., Lauzurica-Gómez S. (2014). Linseed, microalgae or fish oil dietary supplementation affects performance and quality characteristics of light lambs. Span. J. Agric. Res..

[19] Valença R.d.L., da Silva Sobrinho A.G., Borghi T.H., Meza D.A.R., de Andrade N., Silva L.G., Bezerra L.R. (2021). Performance, carcass traits, physicochemical properties and fatty acids composition of lamb’s meat fed diets with marine microalgae meal (*Schizochytrium* sp.). Livest. Sci..

[20] Ponnampalam E.N., Plozza T., Kerr M.G., Linden N., Mitchell M., Bekhit A.E.-D.A., Jacobs J.L., Hopkins D.L. (2017). Interaction of diet and long ageing period on lipid oxidation and colour stability of lamb meat. Meat Sci..

[21] Campo M.M., Nute G.R., Hughes S.I., Enser M., Wood J.D., Richardson R.I. (2006). Flavour perception of oxidation in beef. Meat Sci..

[22] Karabagias I.K. (2018). Volatile Profile of Raw Lamb Meat Stored at 4 ± 1 °C: The Potential of Specific Aldehyde Ratios as Indicators of Lamb Meat Quality. Foods.

[23] De Brito G.F., Holman B.W.B., McGrath S.R., Friend M.A., van de Ven R., Hopkins D.L. (2017). The effect of forage-types on the fatty acid profile, lipid and protein oxidation, and retail colour stability of muscles from White Dorper lambs. Meat Sci..

[24] Gravador R.S., Luciano G., Jongberg S., Bognanno M., Scerra M., Andersen M.L., Lund M.N., Priolo A. (2015). Fatty acids and oxidative stability of meat from lambs fed carob-containing diets. Food Chem..

[25] Insausti K., Murillo-Arbizu M.T., Urrutia O., Mendizabal J.A., Beriain M.J., Colle M.J., Bass P.D., Arana A. (2021). Volatile Compounds, Odour and Flavour Attributes of Lamb Meat from the Navarra Breed as Affected by Ageing. Foods.

[26] Kerth C. (2016). Determination of volatile aroma compounds in beef using differences in steak thickness and cook surface temperature. Meat Sci..

[27] Yang Y., Li J., Jia X., Zhao Q., Ma Q., Yu Y., Tang C., Zhang J. (2022). Characterization of the Flavor Precursors and Flavor Fingerprints in Grazing Lambs by Foodomics. Foods.

[28] Bueno M., Campo M.M., Cacho J., Ferreira V., Escudero A. (2014). A model explaining and predicting lamb flavour from the aroma-active chemical compounds released upon grilling light lamb loins. Meat Sci..

[29] Frank D., Raeside M., Behrendt R., Krishnamurthy R., Piyasiri U., Rose G., Watkins P., Warner R. (2017). An integrated sensory, consumer and olfactometry study evaluating the effects of rearing system and diet on flavour characteristics of Australian lamb. Anim. Prod. Sci..

[30] Wong E., Nixon L.N., Johnson C.B. (1975). Volatile medium chain fatty acids and mutton flavor. J. Agric. Food Chem..

[31] Young O.A., Berdagué J.-L., Viallon C., Rousset-Akrim S., Theriez M. (1997). Fat-borne volatiles and sheepmeat odour. Meat Sci..

[32] Echegaray N., Domínguez R., Cadavez V.A.P., Bermúdez R., Purriños L., Gonzales-Barron U., Hoffman E., Lorenzo J.M. (2021). Influence of the Production System (Intensive vs. Extensive) at Farm Level on Proximate Composition and Volatile Compounds of Portuguese Lamb Meat. Foods.

[33] Grabež V., Bjelanović M., Rohloff J., Martinović A., Berg P., Tomović V., Rogić B., Egelandsdal B. (2019). The relationship between volatile compounds, metabolites and sensory attributes: A case study using lamb and sheep meat. Small Rumin. Res..

[34] Wang F., Gao Y., Wang H., Xi B., He X., Yang X., Li W. (2021). Analysis of volatile compounds and flavor fingerprint in Jingyuan lamb of different ages using gas chromatography–ion mobility spectrometry (GC–IMS). Meat Sci..

[35] Gómez-Cortés P., Sacks G.L., Brenna J.T. (2015). Quantitative analysis of volatiles in edible oils following accelerated oxidation using broad spectrum isotope standards. Food Chem..

[36] Xu C., Zhang S., Sun B., Xie P., Liu X., Chang L., Lu F., Zhang S. (2021). Dietary Supplementation with Microalgae (*Schizochytrium* sp.) Improves the Antioxidant Status, Fatty Acids Profiles and Volatile Compounds of Beef. Animals.

[37] Tansawat R., Maughan C.A.J., Ward R.E., Martini S., Cornforth D.P. (2013). Chemical characterisation of pasture- and grain-fed beef related to meat quality and flavour attributes. Int. J. Food Sci. Technol..

[38] Sivadier G., Ratel J., Bouvier F., Engel E. (2008). Authentication of Meat Products: Determination of Animal Feeding by Parallel GC-MS Analysis of Three Adipose Tissues. J. Agric. Food Chem..

[39] Elmore J.S., Mottram D.S., Enser M., Wood J.D. (2000). The effects of diet and breed on the volatile compounds of cooked lamb. Meat Sci..

[40] Del Bianco S., Natalello A., Luciano G., Valenti B., Monahan F., Gkarane V., Rapisarda T., Carpino S., Piasentier E. (2020). Influence of dietary cardoon meal on volatile compounds and flavour in lamb meat. Meat Sci..

[41] Gkarane V., Brunton N.P., Allen P., Gravador R.S., Claffey N.A., Diskin M.G., Fahey A.G., Farmer L.J., Moloney A.P., Alcalde M.J. (2019). Effect of finishing diet and duration on the sensory quality and volatile profile of lamb meat. Food Res. Int..

[42] Larick D.K., Hedrick H.B., Bailey M.E., Williams J.E., Hancock D.L., Garner G.B., Morrow R.E. (1987). Flavor Constituents of Beef as Influenced by Forage- and Grain-Feeding. J. Food Sci..

[43] Watanabe A., Higechi M. Pastoral Flavour Detection in Beef Fat Using SPME-GCMS. Proceedings of the 50th International Congress of Meat Science and Technology.

[44] Suzuki J., Bailey M.E. (1985). Direct sampling capillary GLC analysis of flavor volatiles from ovine fat. J. Agric. Food Chem..

[45] Buttery R.G., Ling L.C., Teranishi R., Mon T.R. (1977). Roasted lamb fat: Basic volatile components. J. Agric. Food Chem..

[46] Sutherland M.M., Ames J.M. (1995). The effect of castration on the headspace aroma components of cooked lamb. J. Sci. Food Agric..

[47] Hodges K.M., Kerth C.R., Whitney T.R., Wall K.R., Miller R.K., Ramsey W.S., Woerner D.R. (2020). Replacing cottonseed meal and sorghum grain with corn dried distillers’ grains with solubles in lamb feedlot diets: Carcass, trained sensory panel, and volatile aroma compounds traits. J. Anim. Sci..

[48] Kerth C.R., Wall K.R., Miller R.K., Whitney T.R., Stewart W.C., Boles J.A., Murphy T.W. (2019). Effects of feeding juniper as a roughage on feedlot performance, carcass measurements, meat sensory attributes, and volatile aroma compounds of yearling Rambouillet wethers. J. Anim. Sci..

